# Preparation and Properties of CMC-Based Composite Gel as a Flame-Retardant Dust Suppressant

**DOI:** 10.3390/gels12090755

**Published:** 2026-08-24

**Authors:** Jianguo Wang, Zhenzhen Zhang, Xinni He, Binyuan Gao

**Affiliations:** College of Safety Science and Engineering, Xi’an University of Science and Technology, Xi’an 710054, China

**Keywords:** dust suppression, flame retardant, phosphorus–boron synergy, coal spontaneous combustion, wetting property

## Abstract

To address the challenge of balancing flame retardancy and dust suppression in conventional coal mine treatment materials, a multi-component synergistic flame-retardant dust-suppressant gel was fabricated using carboxymethyl cellulose (CMC) as the matrix, compounded with ammonium polyphosphate (APP), zinc borate (ZB), and polycarbodiimide (PCDI) as a cross-linking agent. The optimal formulation was determined via orthogonal experimental design combined with performance characterization, yielding a composition of 1 wt% CMC, 8 wt% APP, 2 wt% ZB, and 0.5 wt% PCDI. Systematic evaluations—including wettability tests, thermogravimetric analysis, and fire-extinguishing trials—demonstrated that the resultant CMC-based composite gel exhibits excellent structural stability and environmental tolerance. Specifically, the contact angle on the coal surface decreased sharply from 72.8° to 17.2°, and the mass loss rate after 30 min of wind erosion was merely 4.16%. Treatment with the gel elevated the critical temperature of the coal–oxygen reaction from 70 °C to 80 °C and reduced CO emissions by 40% at 170 °C. Furthermore, the temperatures corresponding to the maximum weight loss rate, ignition, and burnout increased by 12.9 °C, 16.8 °C, and 28.9 °C, respectively. Fire suppression tests revealed that the gel rapidly cools high-temperature coal seams and effectively prevents reignition. Mechanistic investigations indicate that the CMC-PCDI cross-linked network synergizes with the APP-ZB phosphorus–boron flame-retardant system: the three-dimensional gel architecture provides physical encapsulation and water retention, while the intumescent char layer formed by APP-ZB offers efficient oxygen barrier protection. This study provides a reliable gel-based technical solution for the integrated prevention and control of coal dust pollution and spontaneous combustion disasters in underground mines.

## 1. Introduction

Coal is an important guarantee for China’s energy security, but coal spontaneous combustion fires and dust disasters occur frequently in the process of coal mining, storage and transportation, seriously threatening safe production and green development [1,2,3]. Coal spontaneous combustion not only causes resource loss and environmental pollution, but also easily induces secondary disasters such as gas and coal dust explosion. However, traditional fire prevention and extinguishing technologies have problems such as limited scope of action, poor timeliness and potential to produce secondary pollution [4,5,6]. Coal mine dust endangers the health of workers and increases the risk of coal dust explosion. The existing dust suppression technologies generally have shortcomings such as poor wettability, short action period and single function [7,8,9,10]. With the continuous improvement of coal mine safety and environmental protection requirements, the existing flame-retardant and dust-suppression materials still face problems such as insufficient component synergy, poor penetration stability, and difficulty in balancing flame-retardant and dust-suppression performance [11,12,13,14]. Therefore, it is of great significance to develop a multi-component synergistic composite material with high-efficiency flame retardancy, long-term dust suppression and good environmental adaptability, and to clarify its mechanism of action, to improve the level of coordinated prevention and control of coal mine disasters.

Aiming at the demand for coordinated prevention and control of coal spontaneous combustion and dust disasters in coal mines, domestic and foreign scholars have carried out a lot of research on flame-retardant and dust-suppression materials. Chi et al. [15] combined an inorganic salt inhibitor with a free radical scavenger to prepare a coal spontaneous combustion inhibitor, which realized the synergistic effect of physical barrier and chemical inhibition, and effectively reduced the CO release during coal oxidation. Zhao et al. [16] optimized the PAS-W composite flame-retardant formulation via the response surface method, reducing the content of active functional groups in coal and increasing the ignition temperature. Du et al. [17] used raw materials such as SA, PAAS, ATP and MLT to construct an oxygen-insulating physicochemical synergistic inhibition system, and achieved coal spontaneous combustion inhibition by gel plugging pores and free radical scavenging. The above research mainly focuses on the improvement of coal oxidation inhibition and flame-retardant performance, and pays less attention to the wetting and dust-suppression performance of materials, which cannot meet the needs of collaborative governance of coal mine dust and coal spontaneous combustion disasters. In terms of dust-suppression materials, Ren et al. [18] prepared an environmentally friendly dust-suppression gel with modified straw cellulose as a raw material, which has good wetting, consolidation and biodegradability. Wen et al. [19] constructed a fiber network structure to cooperate with dust-suppression materials, which can improve the ability of coal dust capture and water retention, and delay the oxidation of coal to a certain extent. However, this kind of research mainly focuses on dust control; discussion of flame-retardant performance is relatively limited, and research on the whole-process inhibition mechanism of coal spontaneous combustion is not sufficient. In general, although the existing research has achieved good results in the single field of flame retardancy or dust suppression, there are still various problems: the existing synergistic materials mostly use simple compound systems, the synergistic enhancement mechanism between components is not clear, and the mechanism of material action is not fully understood. In addition, the existing synergistic materials mostly employ simple blending systems, and the synergistic enhancement mechanism between components remains unclear. Therefore, developing CMC-based composite gels with multi-component synergy is significant for addressing these limitations. Such gel systems can leverage their inherent cross-linked networks to achieve the dual functions of dust consolidation and oxygen barrier protection, providing a novel approach for the integrated management of coal mine safety.

Functional materials for coal mine safety are broadly categorized by their fire-retardant mechanisms. Among these, intumescent systems are particularly effective, as they form a protective char layer upon heating to insulate the substrate [20,21]. Conventional intumescent coatings often face limitations such as poor permeability into deep coal seams and susceptibility to cracking under thermal stress. In contrast, gel-type materials offer a distinct advantage by infiltrating porous coal structures and forming an in situ three-dimensional network. This study focuses on a CMC-PCDI composite gel that leverages the phosphorus–boron synergistic chemistry—commonly utilized in advanced intumescent systems—to achieve integrated dust suppression and flame retardancy, thereby addressing the application bottlenecks of traditional coating technologies.

Based on this, this paper uses carboxymethyl cellulose (CMC) as the matrix, introduces ammonium polyphosphate (APP) and zinc borate (ZB) to construct a flame-retardant system, and uses polycarbodiimide (PCDI) as a cross-linking agent to enhance the stability of the material to prepare a multi-component synergistic flame-retardant dust suppressant. Distinct from conventional physically blended suppressants that suffer from component agglomeration and poor performance balance, the novelty of this system lies in the construction of a CMC-PCDI cross-linked network that confines APP and ZB, facilitating their phosphorus–boron synergistic charring effect. The formula was optimized via an orthogonal test, and the optimal system was screened by combining with the penetration depth. The viscosity, wind erosion resistance, water erosion resistance, wetting and dust-suppression effect, and flame-retardant and fire-extinguishing performance were systematically evaluated. The mechanism of multi-component synergistic flame retardancy and dust suppression was revealed, which provides a theoretical basis for the coordinated prevention and control of coal spontaneous combustion and dust disasters in coal mines.

## 2. Results and Discussion

### 2.1. Preliminary Ratio Screening

(1) Orthogonal Experimental Design

Orthogonal experiments were conducted to design nine flame-retardant and smoke-suppressing agents with varying concentrations. Their penetration depths at different time points were measured using a coal pipe column. Experimental results are shown in Table 1.

Among the four factors affecting performance, only CMC and PCDI significantly influence penetration depth, while the other two have minor effects. The order of influence is: PCDI > CMC > APP > ZB. As a water-soluble polymeric modifier, CMC regulates solution viscosity and wettability, balancing permeation resistance and rate while enhancing spreading and penetration capabilities within porous substrates. It also assists cross-linking agent PCDI in constructing cross-linked networks, thereby multidimensionally controlling penetration depth. Synergistic adjustment of PCDI and CMC concentration ratios enables precise optimization of permeation effects.

This study identified four optimal formulations through nine orthogonal experiments, with penetration depth as the core metric: 1% A + 8% B + 2% C + 0.5% D, 1% A + 12% B + 3.5% C + 1.5% D, 1.5% A + 16% B + 3.5% C + 0.5% D, and 2% A + 12% B + 5% C + 0.5% D. These formulations will undergo fundamental performance testing and further optimization.

### 2.2. Results of Basic Performance Tests

#### 2.2.1. Results of Wind Erosion Resistance Tests

**Figure 1:**
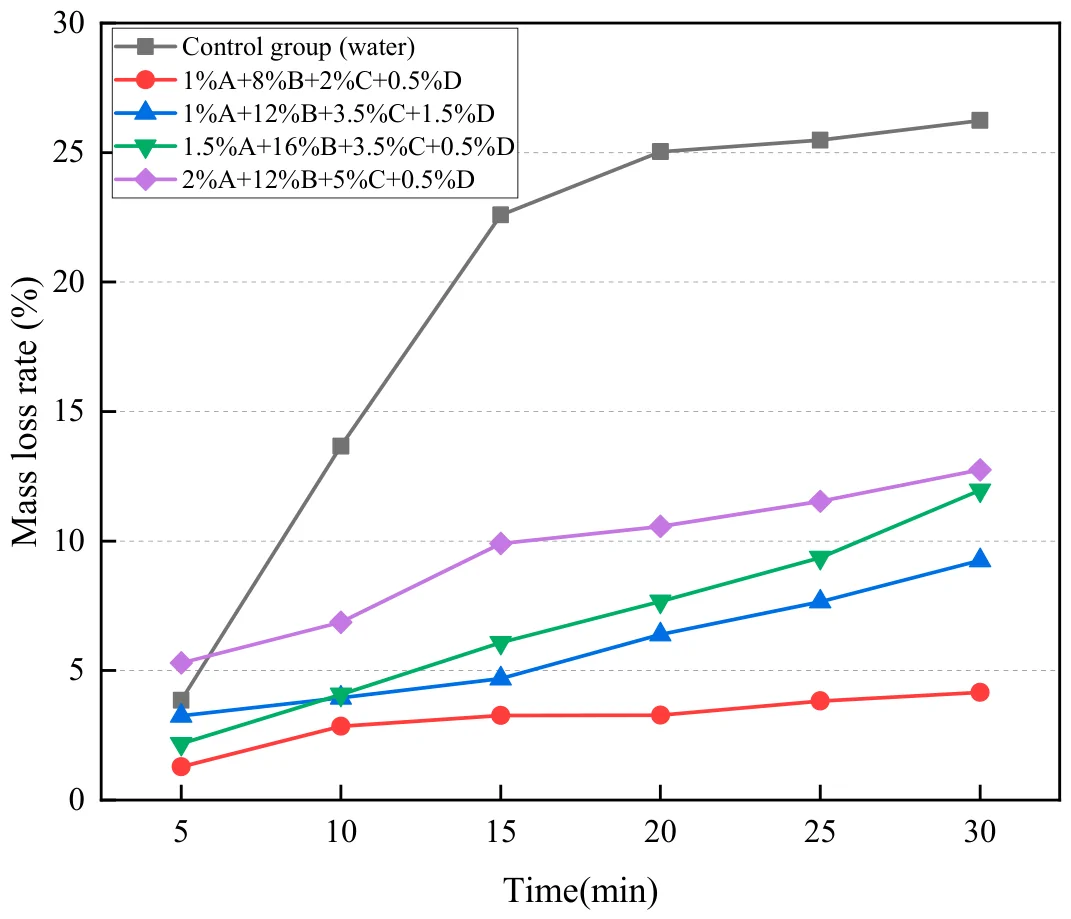
Wind erosion mass loss rate curves for water and flame-retardant dust suppressants at different ratios.

Based on the formula, the experimental data were calculated, and the change trend of the quality loss rate is shown in Figure 1.

The mass loss rates of all experimental groups were significantly lower than the water control group and showed an increasing trend with prolonged wind erosion time. The 1% A + 8% B + 2% C + 0.5% D group exhibited the best wind erosion resistance, with a total mass loss rate of only 4.16% after 30 min, while the 2% A + 12% B + 5% C + 0.5% D group showed a relatively higher rate. The flame-retardant dust suppressant forms a dense consolidation layer on the coal sample surface by constructing a three-dimensional cross-linked network, enhancing mechanical strength and structural stability to effectively resist wind erosion.

#### 2.2.2. Results of Thermal Stability Tests

**Figure 2:**
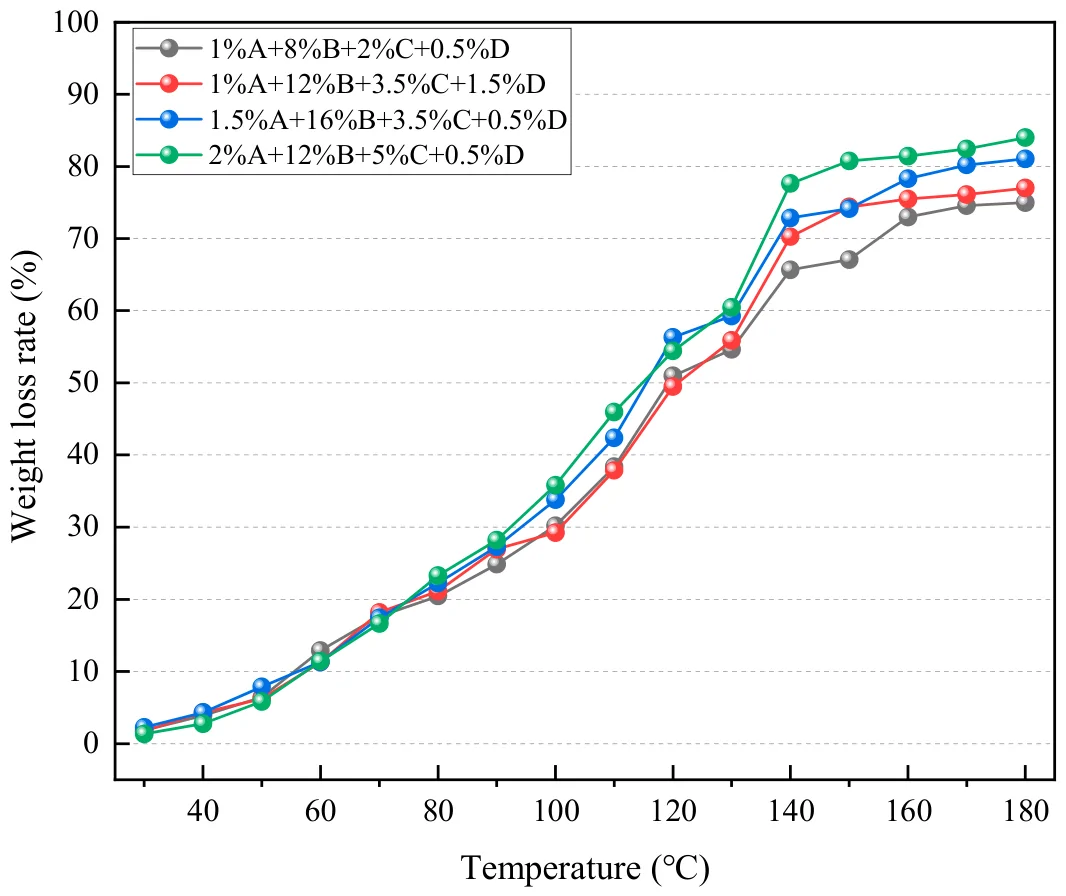
Weight loss curve of flame-retardant dust suppressants under heating conditions.

According to the formula, the mass loss curves of the flame-retardant dust suppressants under programmed heating conditions are presented in Figure 2.

The four groups of samples showed a three-stage weight loss law during the programmed heating process: the weight loss rate was slow in the low temperature range of 20 °C~60 °C, and only the free water and low-boiling-point components were slowly volatilized; in the middle temperature stage of 60 °C~140 °C, the weight loss was rapid, the water was removed in a large amount and the polymer structure was broken. The weight loss rate tended to be stable in the high temperature range of 140 °C~180 °C, and the volatile components were basically removed. Among them, the 1% A + 8% B + 2% C + 0.5% D group had the lowest weight loss rate in the whole temperature range, indicating that its internal consolidation structure was more compact and stable, and its thermal stability was the best.

Based on the results of two basic performance tests, the flame-retardant dust suppressant with the ratio of 1% A + 8% B + 2% C + 0.5% D had the best comprehensive performance and was determined as the research object of subsequent experiments.

### 2.3. Results of Dust-Suppression Performance Tests

#### 2.3.1. Results of Contact Angle Measurements

**Figure 3:**
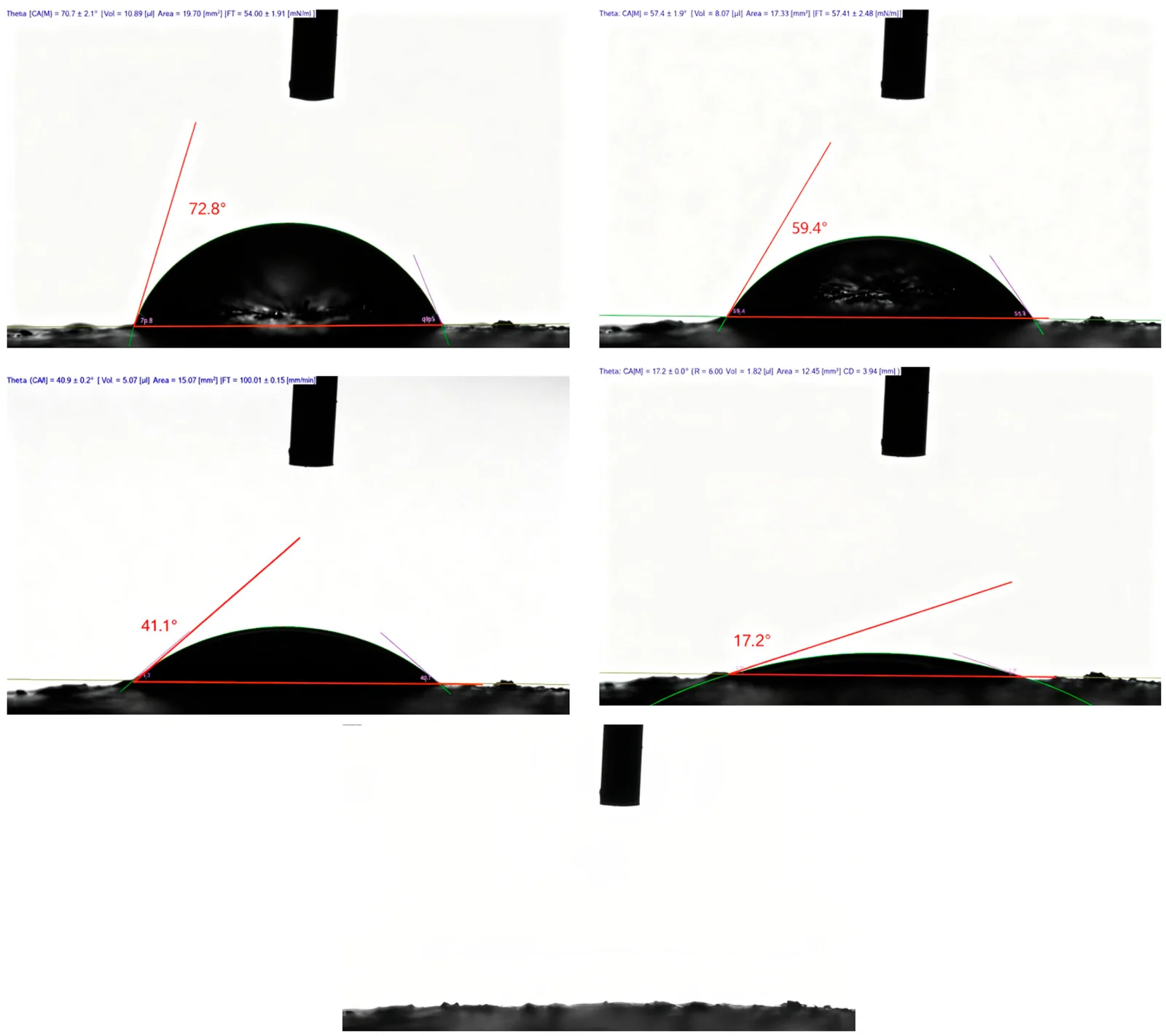
The change in contact angle morphology during the wetting process.

The contact angle is determined by the balance of liquid cohesion and solid–liquid adhesion [22,23]. The smaller the angle is, the better the wettability is. The wetting of the coal seam is divided into three stages: wetting, spreading and wetting. The change in contact angle is shown in Figure 3.

The initial time (0 s) of the dust-suppressant solution in Figure (**a**) shows that the droplet is in a typical Wenzel state on the surface of the coal sheet, and the contact angle is 72.8° (CAIM = 70.7 ± 2.1°), indicating that there is a hydrophobic microporous structure on the surface of the coal. With the passage of time (**b**–**d**), the droplets completed rapid spreading within 0.3 s (the contact angle decreased from 72.8° to 17.2°), and the spreading rate (dθ/dt ≈ 184°/s) was significantly higher than that of conventional wetting agents. Figure (**e**) shows that the droplets are completely infiltrated and the contact angle disappears at 0.4 s, which proves that the solution has ultra-fast wetting kinetic characteristics. The dust-proof mechanism is the rapid adsorption of active components on the surface of coal dust and the reconstruction of interface energy. The molecules migrate rapidly to the solid–liquid interface to reduce the interfacial tension, thus driving the droplets to spread and penetrate into the dust pores. After the spreading is completed, the solution forms a dense hydrophilic wetting film on the surface of the coal body. By eliminating the concave contour of the liquid bridge, the dust is quickly wrapped and coagulated to achieve efficient dust suppression.

#### 2.3.2. Results of Dynamic Surface Tension Measurements

**Figure 4:**
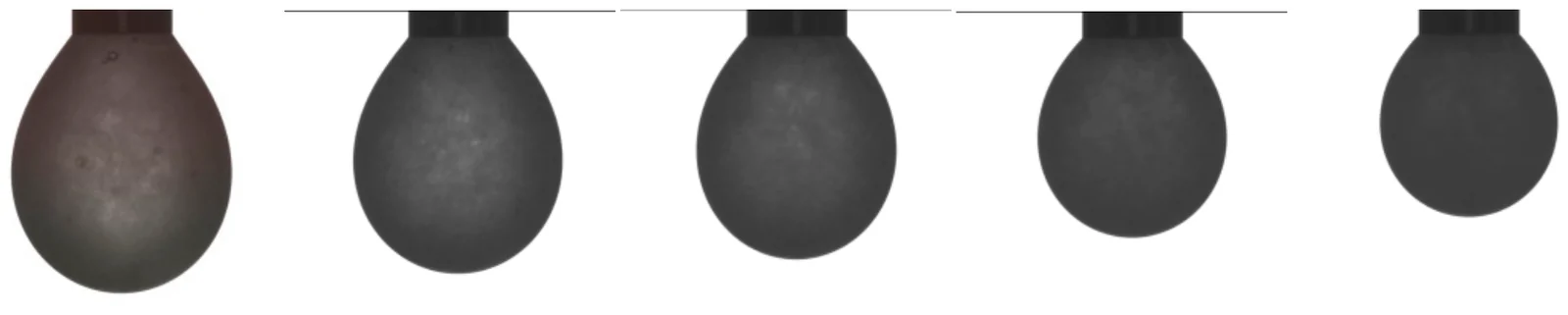
Flame-retardant dust-suppressant droplet morphology change diagram.

In this test, the side profile image of the droplet of the flame-retardant dust-suppressant solution changing with time is captured by the hanging drop method, as shown in Figure 4.

**Figure 5:**
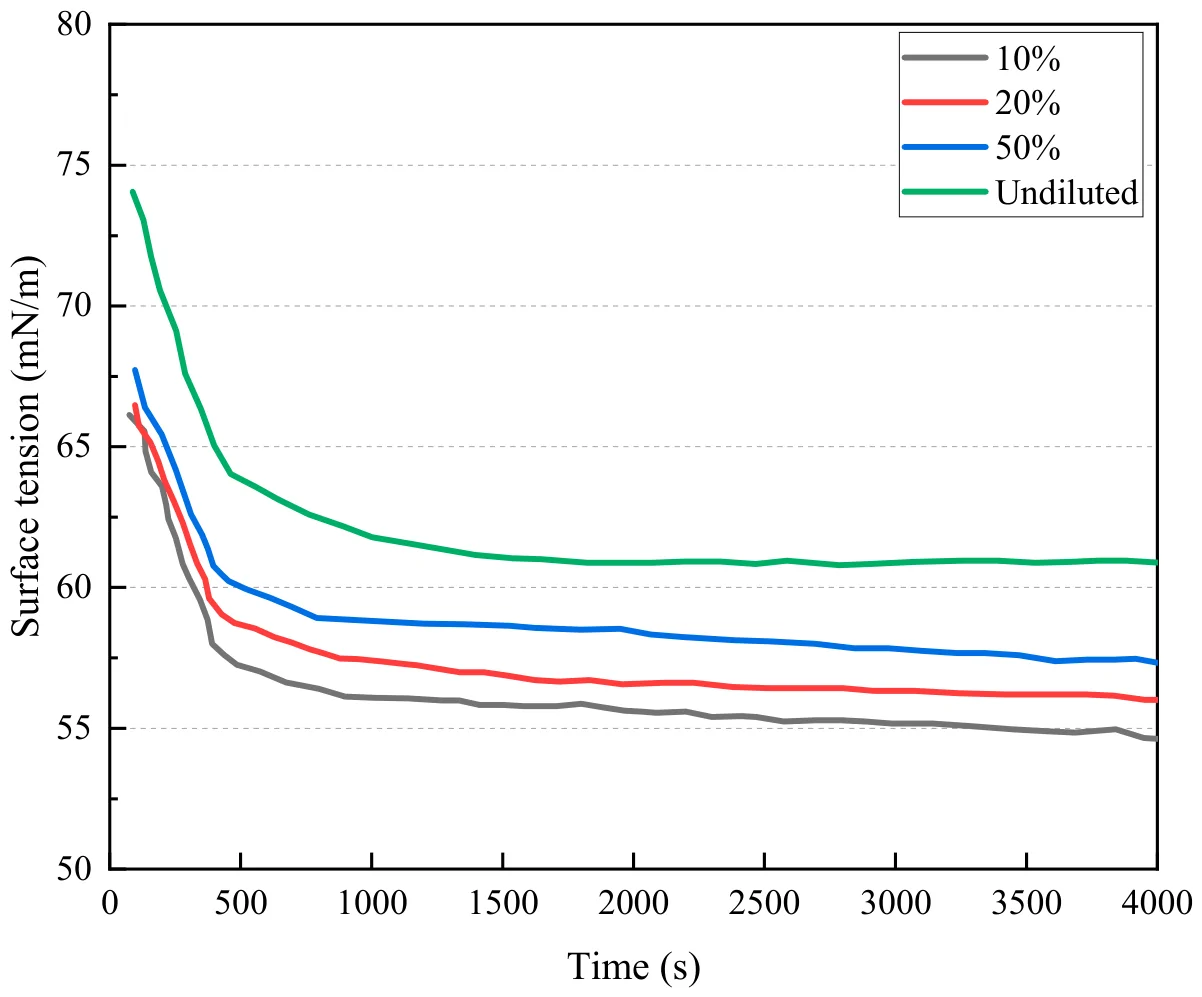
Contact angle change curve during the wetting process.

Through the dynamic surface tension measurement experiment, the variation in the surface tension of the dust-suppressant solution with time under the undiluted stock solution and 50%, 20% and 10% dilution concentrations was systematically investigated. The results are shown in Figure 5.

The surface tension of the four diluted concentrations of dust suppressants showed the characteristics of “initial rapid drop and later stability” and fluctuated slightly. The active components were enriched in 0~1000 s due to solute concentration, and the interface adsorption reached equilibrium after 1000 s. The surface tension is positively correlated with the concentration. The initial and stable values are undiluted > 50% > 20% > 10%, which reflects the concentration effect and dynamic adsorption process of the system. The dust suppressant can reduce the surface tension at all concentrations, and the interface activity is stable. It has excellent wetting, spreading and permeability to hydrophobic coal dust. It can efficiently wet agglomerated fine coal dust in a wide concentration range and achieve stable and reliable consolidation dust suppression.

### 2.4. Results of Flame Retardancy Tests

#### 2.4.1. Fourier Transform Infrared (FTIR) Spectroscopy Analysis

**Figure 6:**
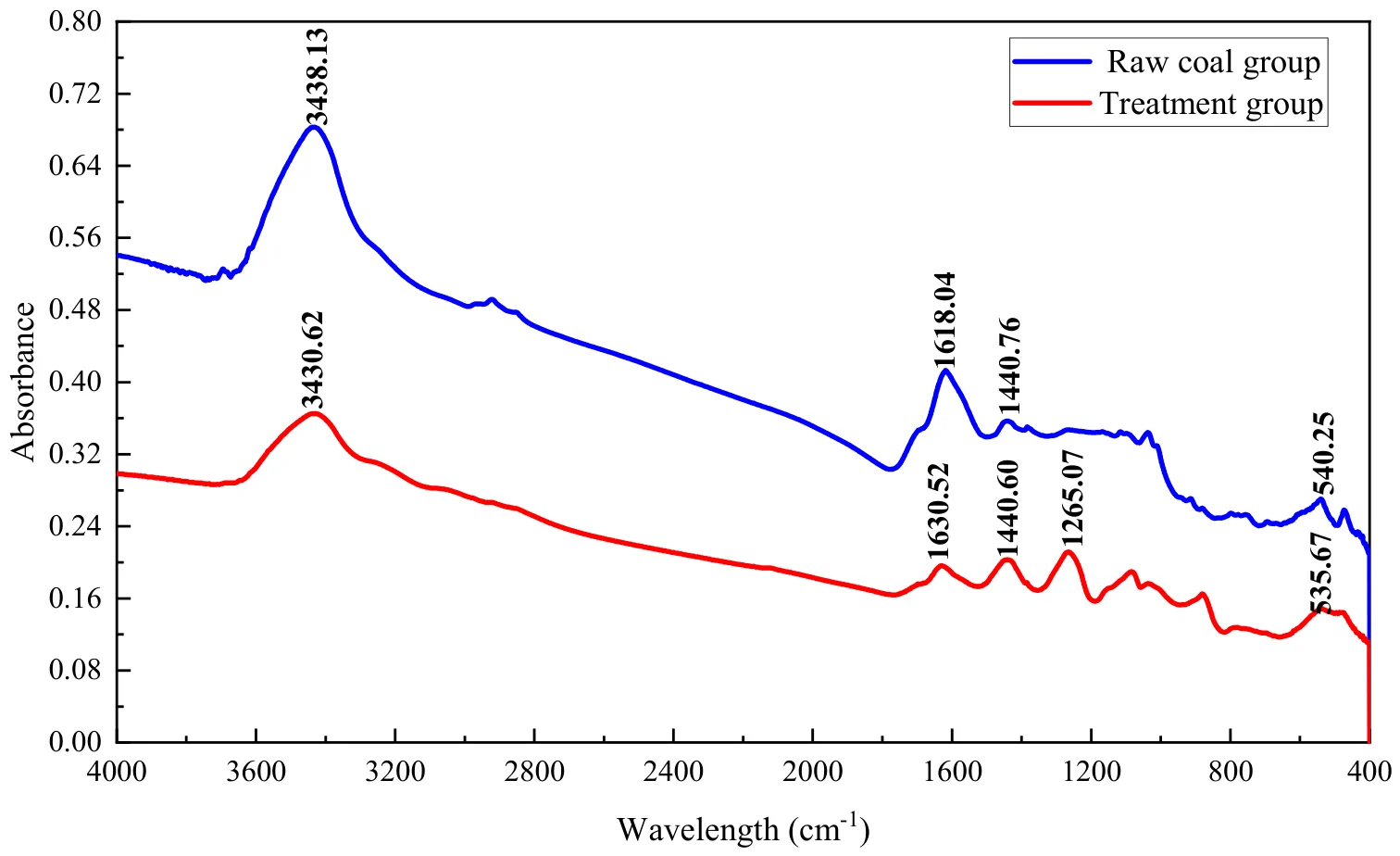
Infrared absorption spectra of different samples.

The infrared absorption spectra of different samples are shown in Figure 6.

As shown in Figure 6, the infrared spectral curves of raw coal and flame-retardant–dust-suppressant-treated coal samples are similar as a whole. The absorbance of the treatment group is generally low, and the position and intensity of some characteristic peaks are obviously changed. The hydroxyl peak at 3600~3200 cm^−1^ shifted from 3438.13 cm^−1^ to 3430.62 cm^−1^, indicating that the flame retardant and coal hydroxyl formed hydrogen bonds and other interactions, which improved the binding force and stability of the gel and coal. The peak of carbonyl/carbon–carbon double bond at 1800~1500 cm^−1^ shifted from 1618.04 cm^−1^ to 1630.52 cm^−1^, and the chemical environment of functional groups changed. The peak intensity of methyl, methylene and carbon–oxygen single bond at 1455~1000 cm^−1^ was significantly weakened. The characteristic peak of 540.25 cm^−1^ at 900~400 cm^−1^ shifted to 535.67 cm^−1^, which further confirmed the interaction between functional groups. The coal mainly contains functional groups such as hydroxyl, carbonyl, carbon–carbon double bond, methyl, methylene and carbon–oxygen single bond. The flame retardant and dust suppressant can effectively inhibit the coal–oxygen reaction by changing the displacement and intensity of the characteristic peak by interacting with these functional groups.

#### 2.4.2. Analysis of Mass and Heat Variation Characteristics

**Figure 7:**
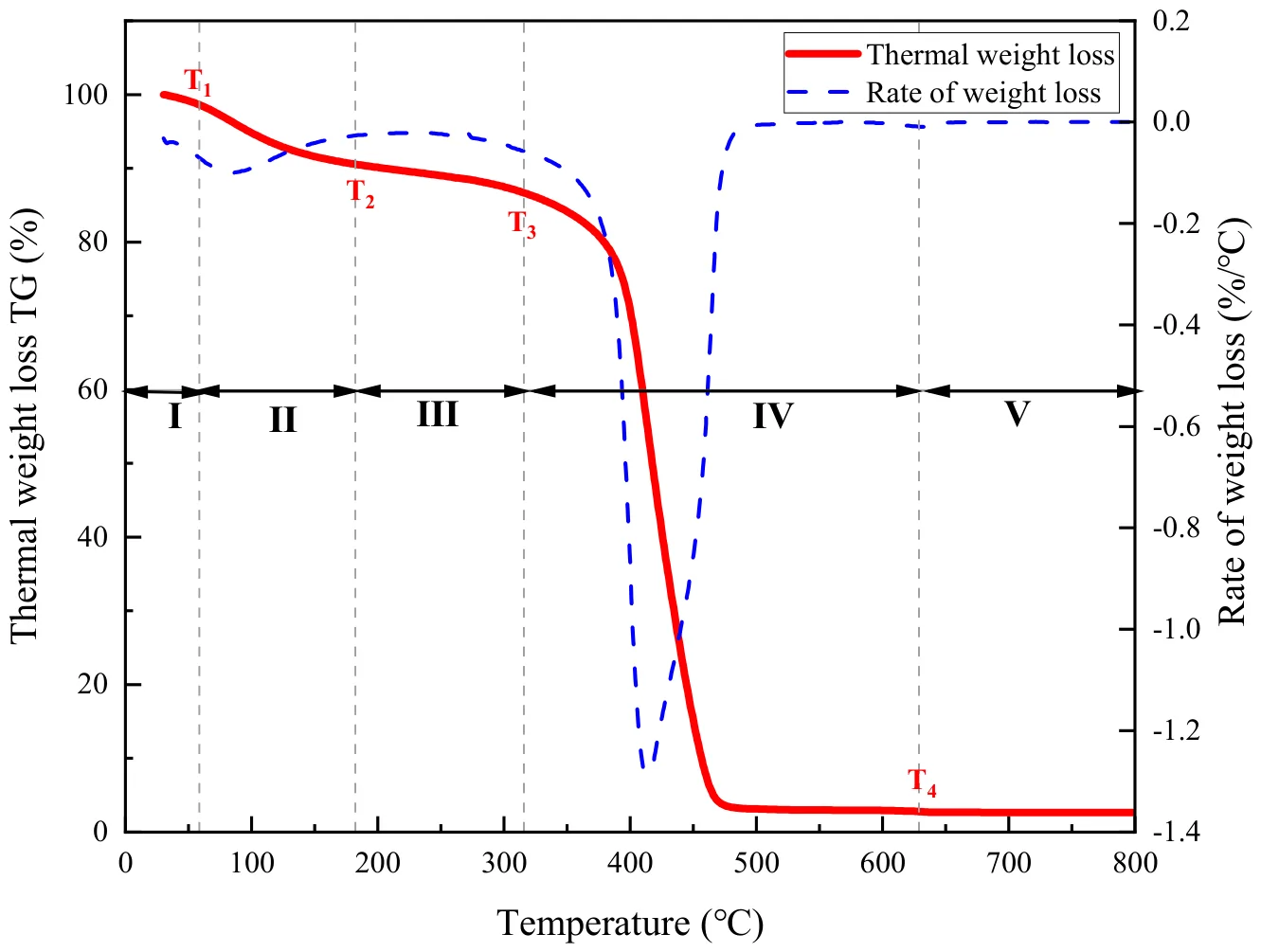
TG-DTG curve of raw coal group coal sample.

**Figure 8:**
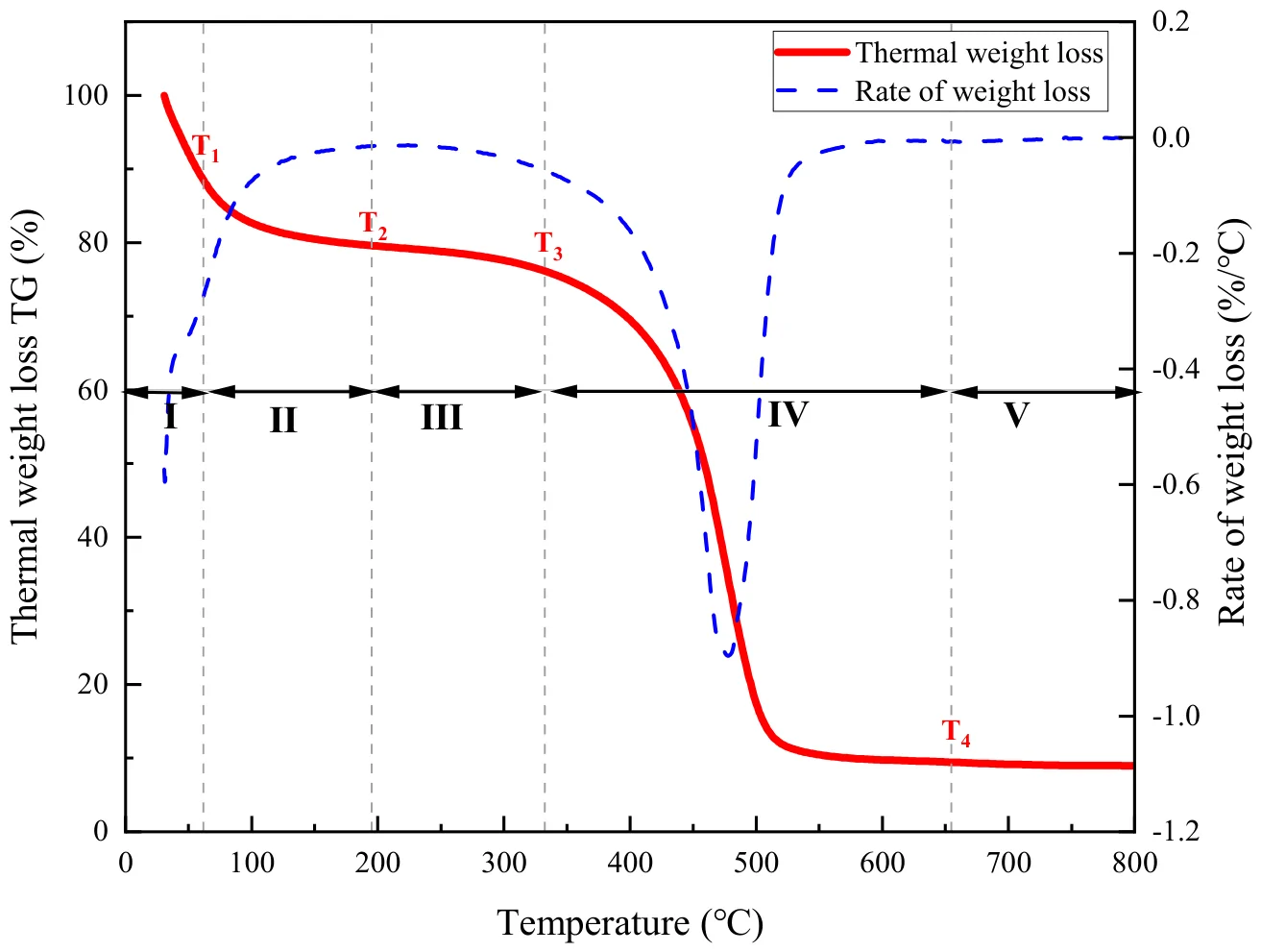
TG-DTG curve of coal samples treated with flame retardant and dust suppressant.

Through the thermogravimetric analysis of raw coal and treated coal samples, the inhibition effect of the flame-retardant dust suppressant on coal spontaneous combustion is judged. The TG, DTG curves and main parameters are shown in Figure 7 and Figure 8.

Combined with the thermal weight loss curve of Figure 7 and Figure 8, it can be seen that the combustion process of raw coal and flame-retardant-treated coal samples is divided into five stages—initial weight loss, oxygen absorption weight gain, thermal decomposition, combustion, and burnout—and the overall thermal weight loss trend is basically the same. However, the addition of flame retardants significantly delayed the characteristic temperature of each stage, and the weight loss curve was more gentle, showing a good flame-retardant delay effect. The specific mechanism is as follows: In the initial weight loss stage, the flame retardant delays the initial evaporation temperature T1 (61.8 °C) by 3.3 °C compared with the raw coal group (58.5 °C) by adsorbing gas and locking water. In the stage of oxygen absorption and weight gain, the flame retardant hinders the contact between coal and oxygen, delays the oxidation process, and delays the maximum weight temperature T2 (195.2 °C) by 12.9 °C compared with the raw coal group (182.3 °C). In the thermal decomposition stage, the flame retardant inhibited the pyrolysis process, and the ignition temperature T3 (332.5 °C) increased by 16.8 °C compared with the raw coal group (315.7 °C). In the combustion stage, the flame retardant increases the temperature required for the complete reaction of the combustible material, so that the burnout temperature T4 (657.8 °C) is 28.9 °C higher than that of the raw coal group (628.9 °C). In the burnout stage, the residual mass of the flame-retardant treatment group was slightly higher than that of the raw coal group, which may be due to the incomplete decomposition of its components or the formation of stable ash by interaction with minerals in coal.

#### 2.4.3. Flame-Retardant Dust Suppressant Fire Performance

The coal samples used in the test were taken from the original coal seam of Shenmu Chuangwei Coal Mine. The sampling process was strictly in accordance with the provisions of the GB/T 19222-2003 [24] “coal rock sample taking method” for bituminous coal, and the size of the coal blocks was not less than 10 cm × 5 cm × 5 cm. After the coal sample was collected, it was immediately put into a sample bag and sealed to prevent oxidation. Subsequently, the industrial analysis of the original coal sample was carried out according to GB/T 212-2008 [25] “Industrial analysis method of coal”, and the results are shown in Table 2.

**Figure 9:**
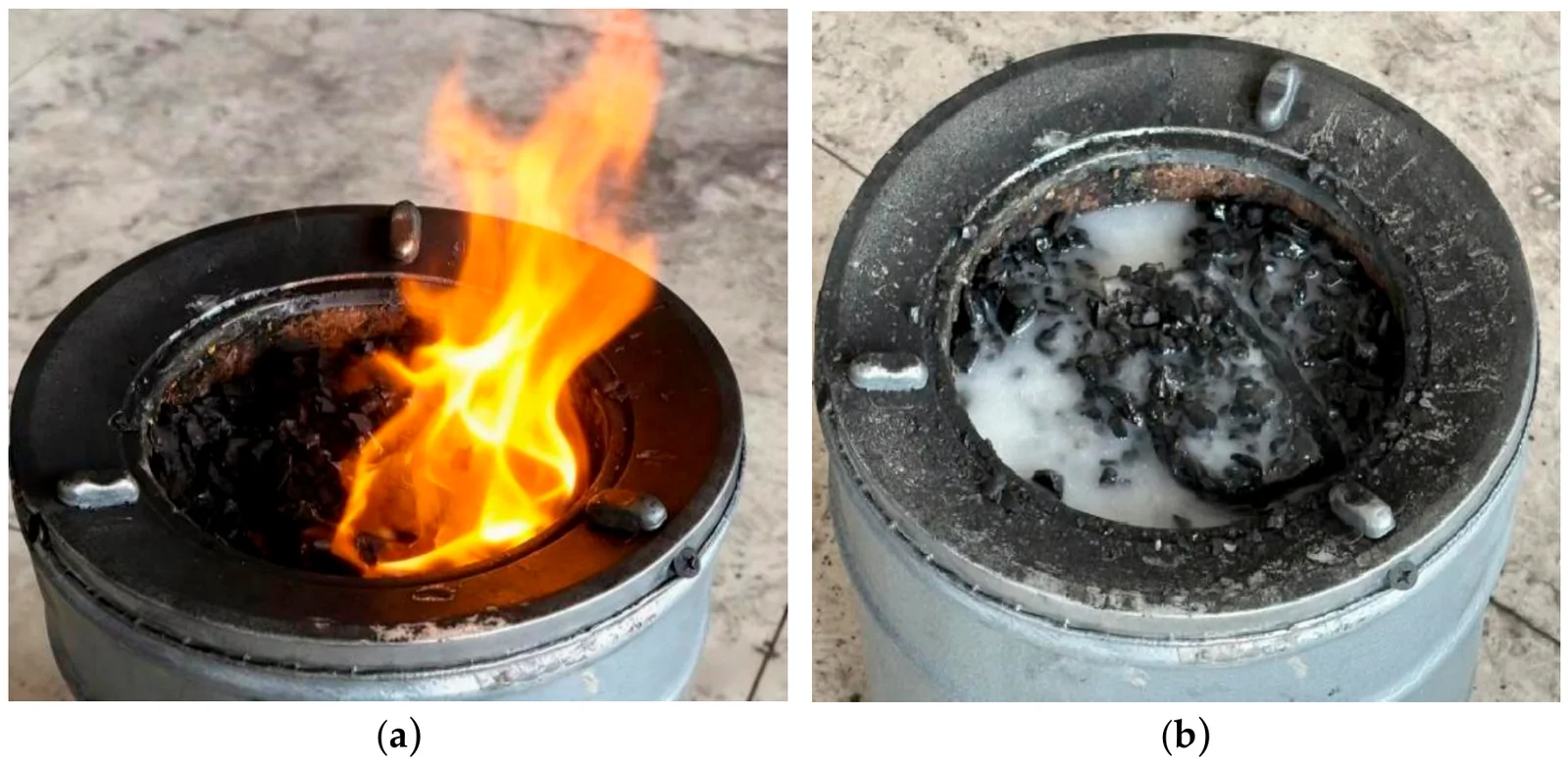
Comparison diagram of flame-retardant dust suppressant before and after fire extinguishing. (**a**) Before adding flame-retardant dust suppressant; (**b**) after adding flame-retardant dust suppressant.

**Figure 10:**
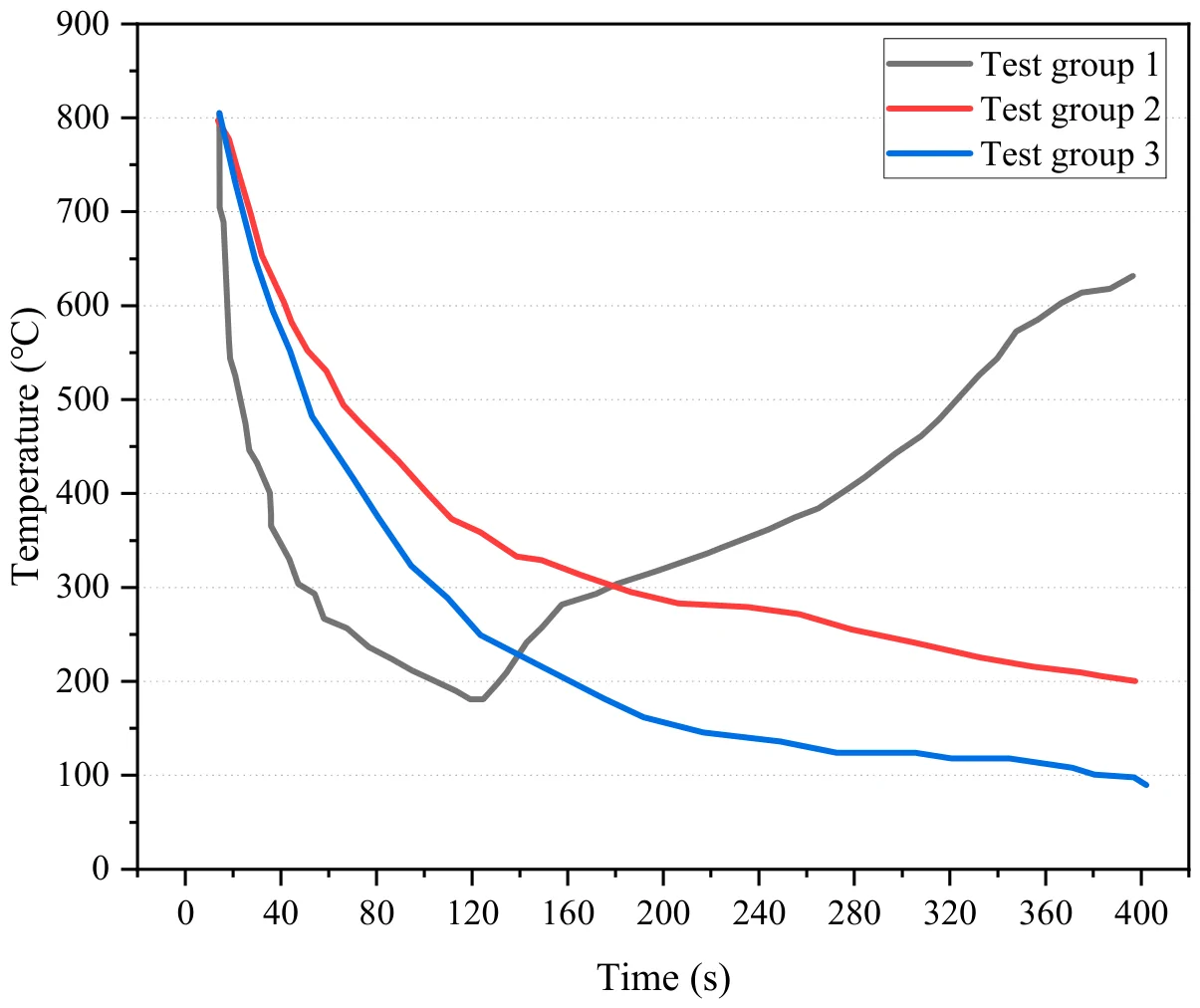
Temperature curve of fire-extinguishing experiment.

The flame retardant prepared in this paper was added before and after the fire extinguishing as shown in Figure 9, and the temperature curve of the fire-extinguishing experiment is shown in Figure 10.

It can be clearly seen from Figure 10 that during the test period of 0~400 s, the temperature changes of the three groups of coal bodies show obvious differences: the temperature of test group 1 began to decrease rapidly from about 800 °C, and the temperature decreased significantly due to the rapid penetration of water into the coal seam in the early stage, but it began to rise continuously after falling to about 180 °C at about 100 s, and rose to more than 600 °C at 360 s. There was a significant reburning phenomenon, which was due to the gradual evaporation of water and the loss of oxygen insulation and cooling effect of water in coal. The data of test groups 2 and 3 showed that after adding the flame retardant with a ratio of 1% A + 8% B + 2% C + 0.5% D, the coal temperature began to decrease at the fastest rate from 800 °C, and the rate of decline slowed down after 120 s, and finally stabilized at a low level of about 100 °C. There was no reburning phenomenon of temperature recovery in the whole process, indicating that the new flame-retardant dust suppressant could continue to play the role of oxygen insulation and cooling. Overall, its cooling and anti-reburning effects were significantly better than that of water.

#### 2.4.4. Analysis of Flame-Retardant Mechanism

(1) Synergistic Water Retention–Cooling and Flame-Retardant Dispersion via Gel Network

The CMC-PCDI cross-linked gel network serves as the matrix, featuring a porous three-dimensional architecture formed under the action of the cross-linking agent. This gel network not only locks in substantial amounts of water to enable continuous endothermic cooling and dust consolidation but also uniformly disperses APP and ZB within its interstitial spaces, effectively preventing additive agglomeration and laying a robust structural foundation for synergistic flame retardation. Concurrently, the physical encapsulation of coal dust by the porous gel scaffold further blocks the pathways for dust involvement in combustion, achieving integrated dust suppression and preliminary fire prevention.

(2) Phosphorus–Boron Synergistic Char Formation and Efficient Oxygen Barrier Plugging

During heating, the synergistic interaction between APP and ZB yields a dense, stable composite char layer: APP catalyzes dehydration and carbonization to form an initial carbonaceous matrix, while ZB releases crystal water for evaporative cooling and simultaneously fills micropores within this matrix. Collectively, they construct a crack-free, compact oxygen-barrier gel-derived char layer. This char layer integrates tightly with the underlying CMC-PCDI cross-linked gel network, effectively sealing fissures in the coal body to realize dual oxygen isolation via “char layer barrier + surface densification.” The oxygen-blocking efficiency of this gel-based system significantly surpasses that of single-component flame retardants.

(3) Inhibition of Active Groups and Reduction in Spontaneous Combustion Tendency

The acidic species generated from APP decomposition catalyze the dehydration and cross-linking of oxygen-containing functional groups in coal, thereby reducing the yield of combustible volatiles. Decomposition products of ZB selectively capture active free radicals, interrupting the combustion chain reaction at the molecular level. Furthermore, the stable phosphorus–boron complexes formed synergistically adsorb onto active sites of the coal matrix, passivating the reactivity of coal macromolecules. This process elevates the activation energy for ignition and delays the onset of thermal runaway, fundamentally diminishing the spontaneous combustion propensity of treated coal seams.

**Figure 11:**
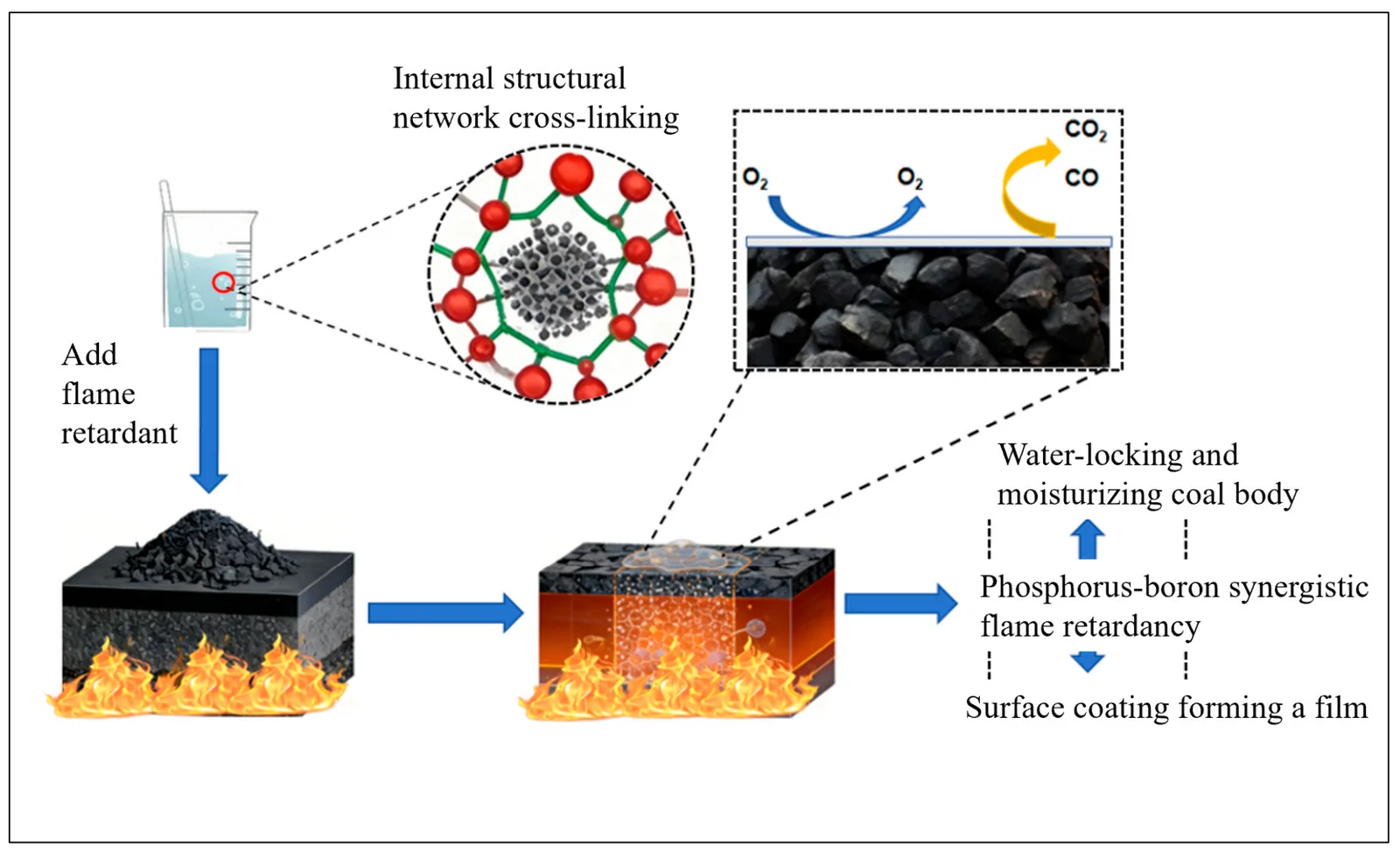
Flame-retardant dust-suppressant mechanism diagram.

### 2.5. Comparative Analysis with Existing Suppressants

To further highlight the advancement of the proposed CMC-PCDI-APP-ZB gel over conventional single-function or physically blended suppressants, a comparative analysis was conducted, as summarized in Table 3.

## 3. Conclusions

(1) Formulation and Comprehensive Performance of the Composite Gel

This study constructed a CMC-based composite flame-retardant dust-suppressant gel via APP/ZB/PCDI synergy. Through orthogonal experimental design coupled with comprehensive performance validation, the optimal formulation was determined as 1 wt% CMC, 8 wt% APP, 2 wt% ZB, and 0.5 wt% PCDI. This gel system exhibited exceptional multifunctional performance: the contact angle on the coal surface decreased dramatically from 72.8° to 17.2°, achieving complete droplet spreading within 0.3 s. Furthermore, the mass loss rate after 30 min of wind erosion was merely 4.16% ± 0.32%, significantly lower than that of the high-content control group (7.89% ± 0.45%). During programmed heating from 30 to 160 °C, the gel maintained the lowest weight loss rate throughout the process, demonstrating outstanding structural stability and environmental tolerance, attributed to the robust CMC-PCDI cross-linked network.

(2) Synergistic Enhancement of Wetting and Flame Retardancy

The CMC-based gel achieves a synergistic enhancement of dust suppression via wetting and fire retardation via inhibition. Regarding wetting performance, the dynamic surface tension of the gel precursor solution reached a stable low value within 1000 s across all concentration gradients, indicating excellent permeability and encapsulation capabilities for hydrophobic coal dust. Regarding flame retardancy, the combined action of the phosphorus–boron synergistic system and the three-dimensional gel network elevated the critical temperature of the coal–oxygen reaction from 70 °C to 80 °C. At 170 °C, CO emissions were reduced by 26.9% (5% additive group) and 40.0% (10% additive group) compared to raw coal. Thermogravimetric analysis revealed that the temperatures corresponding to the maximum mass loss rate, ignition, and burnout were increased by 12.9 °C, 16.8 °C, and 28.9 °C, respectively, effectively delaying the spontaneous combustion process of coal.

(3) Action Mechanisms and Implications

The multifunctional mechanism of the proposed gel can be summarized in three aspects: First, the water retention and physical encapsulation effect of the CMC-PCDI gel network delayed the onset temperature of moisture evaporation by 3.3 °C compared to raw coal. Second, the phosphorus–boron synergistic char formation; the APP-ZB system formed a dense, crack-free gel-derived char layer that effectively isolated oxygen, exhibiting superior barrier efficiency compared to single-flame-retardant systems. Third, the active radical scavenging and functional group passivation effect. Fourier transform infrared (FTIR) spectroscopy confirmed specific interactions between the gel and active functional groups in coal, leading to significant alterations in the chemical microenvironment and reducing the oxidation activity at the source. These findings provide a reliable gel-based technical solution for the integrated prevention and control of compound disasters involving coal dust and spontaneous combustion in underground mines.

## 4. Materials and Methods

### 4.1. Experimental Materials and Equipment

The test materials include: sodium carboxymethyl cellulose (CMC, 99%, China Pharmaceutical Group Chemical Reagents Co., Ltd., Shanghai, China); ammonium polyphosphate (APP, 99%, Ruiyuan Chemical, Zhejiang, China); zinc borate (ZB, 99%, Tianjin Huasheng Chemical Reagent Co., Ltd., Tianjin, China); polycarbodiimide (PCDI, 99%, Guangzhou Wanjun Chemical Technology Co., Ltd., Guangzhou, China); and deionized water.

The experimental equipment includes an electronic balance (0.01 g), a laboratory electric stirrer (LC-ES-120), a dial-type rotational viscometer and blower drying oven. The electronic balance, laboratory electric stirrer, and blower drying oven were all purchased from Lychen Technology Co., Ltd. (Zhejiang, China).

### 4.2. Sample Preparation

**Figure 12:**
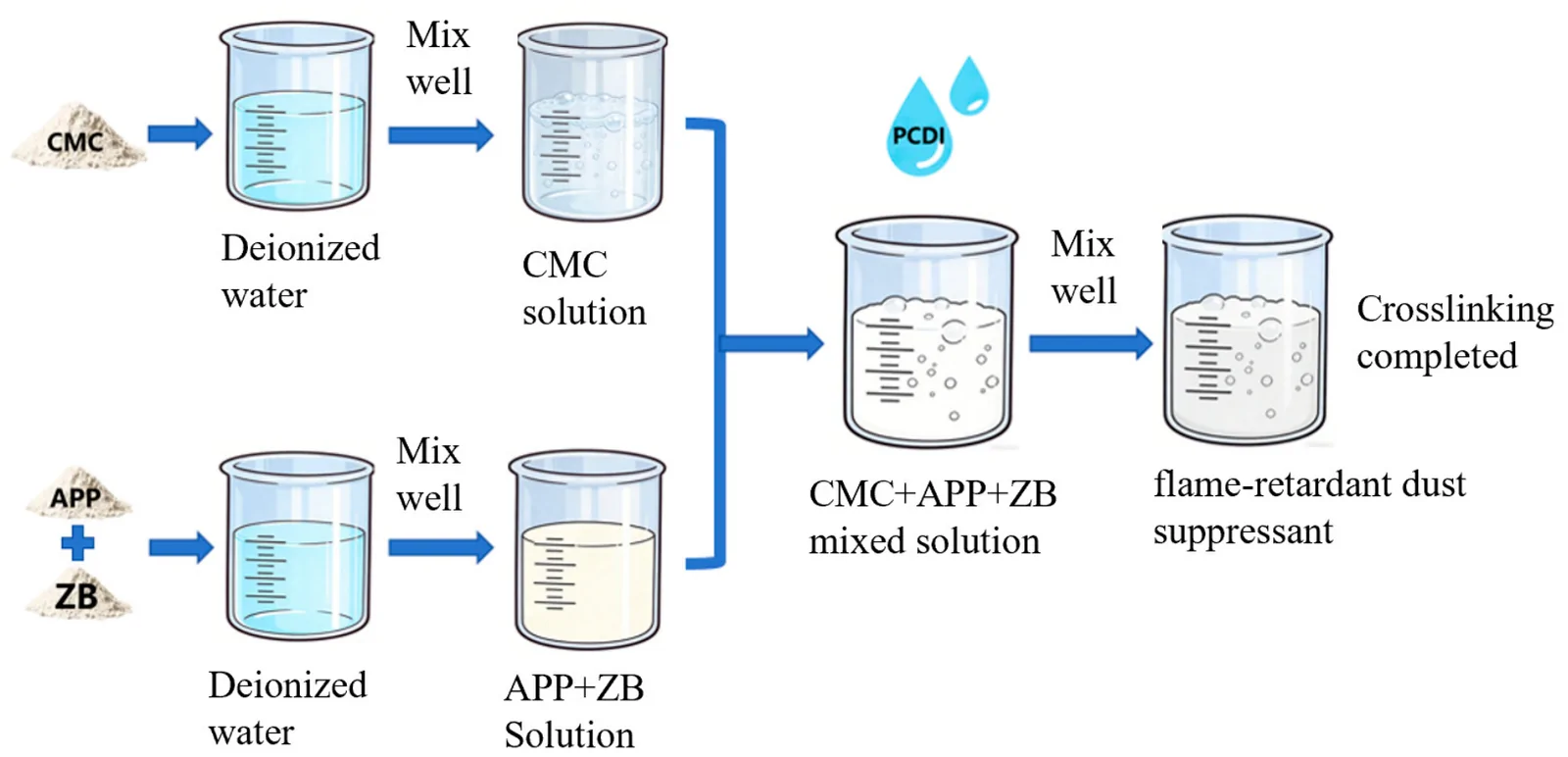
Schematic illustration of the sample preparation process.

Sodium carboxymethyl cellulose (CMC) was first dissolved in deionized water and stirred at 40–50 °C until completely solubilized to prepare a 1–2 wt% CMC stock solution. Subsequently, ammonium polyphosphate (APP) and zinc borate (ZB) were weighed according to the mass ratio of CMC:APP:ZB = 1:12:3.5 and dispersed in the remaining deionized water under high-speed stirring to obtain a homogeneous composite dispersion. This dispersion was then added dropwise to the CMC solution under moderate agitation to yield a premix. Finally, polycarbodiimide (PCDI) cross-linker, accounting for 1–5 wt% of the total solid content, was introduced into the premix at room temperature or 40–60 °C with continuous stirring. After the addition was completed, the mixture was stirred for an additional 30–60 min to ensure thorough homogeneity and allowed to stand for 12–24 h to afford the novel flame-retardant dust-suppressant gel, during which the CMC-PCDI cross-linked network was fully developed. A schematic illustration of the sample preparation process is presented in Figure 12.

### 4.3. Basic Performance Characterization

#### 4.3.1. Wind Erosion Resistance Test

Approximately 300 g of standard coal powder (particle size: 0–0.9 mm) was weighed and spread evenly in a tray. Subsequently, about 100 g of the composite flame-retardant dust suppressant was sprayed uniformly onto the coal powder surface to ensure adequate penetration. The samples were air-dried naturally until a complete solidified crust formed. The initial mass (m_0_) was recorded. A 30 kW air blower was then employed, with its outlet positioned 20 cm above the coal sample surface. The sample was subjected to a constant wind speed for 30 min. The mass was measured at intervals of 5, 10, 15, 20, 25, and 30 min during the erosion process. The mass loss rate (Q_R_) was calculated according to Equation (1) to evaluate the wind erosion resistance:

(1) $$Q_{R}=\frac{m_{0}-m_{1}}{m_{0}}\times 100\%$$

where $Q_{R}$—mass loss rate, %; $m_{0}$—initial mass of the coal sample after spraying and drying, g; and $m_{1}$—mass of the coal sample after wind erosion for 5, 10, 15, 20, 25, and 30 min, respectively, g.

#### 4.3.2. Thermal Stability Test

Approximately 100 g of the dust-suppressant samples prepared according to the four optimized formulations were weighed and placed into pre-weighed weighing bottles, respectively. The samples were then subjected to a programmed heating regime in a constant-temperature blast drying oven to evaluate their thermal stability. The temperature program was set as follows: heating commenced at 30 °C, followed by an increase of 10 °C every 30 min until reaching 160 °C. At each designated temperature point (i.e., at 30 min intervals), the samples were removed, rapidly cooled to room temperature, and weighed. The mass loss rate (*W_L_*) within each temperature interval was calculated according to Equation (2):

(2) $$W_{L}=\frac{m_{0}-m_{i}}{m_{0}}\times 100\%$$

where $W_{L}$—mass loss rate, %; $m_{0}$—initial mass of the flame-retardant dust-suppressant sample, g; and $m_{i}$—mass of the sample at a specific time point, g.

### 4.4. Dust-Suppression Performance Test

#### 4.4.1. Contact Angle Measurement

The contact angle serves as a pivotal indicator for evaluating the dust-suppression performance of materials, as its value defines the surface wettability type of solids [26]. In this study, a German LAUDA Scientific LSA100S optical contact angle measuring system was employed to determine the contact angles of the optimized flame-retardant dust suppressants. This analysis characterizes the wettability and spreading behavior of the suppressants on the coal dust surface, thereby elucidating the underlying dust suppression mechanism.

Approximately 500 mg of dried coal powder (particle size: 0–0.9 mm) was placed into a tablet press mold and compressed at 30 MPa for 5 min to fabricate a smooth, circular pellet. The prepared coal pellet was horizontally fixed on the sample stage of the instrument. A microsyringe was used to dispense a 2–5 µL droplet of the test solution onto the pellet surface. Within 0.5 s of droplet deposition, the contour image of the sessile drop was rapidly captured, and the contact angle was subsequently analyzed and calculated using the instrument’s proprietary software.

#### 4.4.2. Dynamic Surface Tension Measurement

The wetting performance of materials critically depends on kinetic processes, and dynamic surface tension serves as a core indicator for efficient dust capture [27,28,29,30]. In this study, the pendant drop method integrated with a contact angle goniometer was employed to measure surface tension. This approach ensures complete equilibration, facilitates the investigation of liquid aging effects, and offers convenient data processing with minimal experimental error.

A stock solution and its diluted variants (50%, 20%, and 10% by concentration) were prepared and subjected to ultrasonic degassing prior to being transferred into syringes. The surface tensiometer was preheated and stabilized at 25 °C for 30 min, and the density difference parameters were input into the system. Subsequently, the syringe was mounted onto the sample stage to form a pendant droplet. Starting from the time zero, droplet images were captured at 5 s intervals over a duration of 4000 s. The acquired images were analyzed using dedicated software for curve fitting, enabling the calculation and output of surface tension values at each time point, thereby yielding the dynamic surface tension curves.

### 4.5. Flame Retardancy Test

#### 4.5.1. Infrared Spectroscopy Measurement

Fourier transform infrared (FTIR) spectroscopy was employed to characterize the functional groups of both the composite material and the coal samples, with the aim of elucidating the correlation between the functional groups of the flame-retardant dust suppressant and its performance, as well as revealing the underlying mechanism by which it inhibits the coal–oxygen reaction. A raw coal control group and a suppressant-treated test group were established. Potassium bromide (KBr) was thoroughly dried, mixed with the samples at a mass ratio of 1:100, and pressed into pellets. FTIR spectra were subsequently recorded over a wavenumber range of 4000–400 cm^−1^ with 32 scans per sample.

#### 4.5.2. TG-DSC Measurements

Simultaneous TG-DSC analysis was performed using a simultaneous thermal analyzer. Raw coal and coal samples treated with the flame-retardant dust suppressant were dried at 35 °C for 24 h, ground, and passed through a 100-mesh sieve. Approximately 10 mg of each sample was placed in an alumina crucible and subjected to synchronous measurement from 30 to 800 °C at a heating rate of 5 K·min^−1^ under a nitrogen atmosphere with a constant flow rate of 50 mL·min^−1^.

#### 4.5.3. Fire-Extinguishing Experiment Test

**Figure 13:**
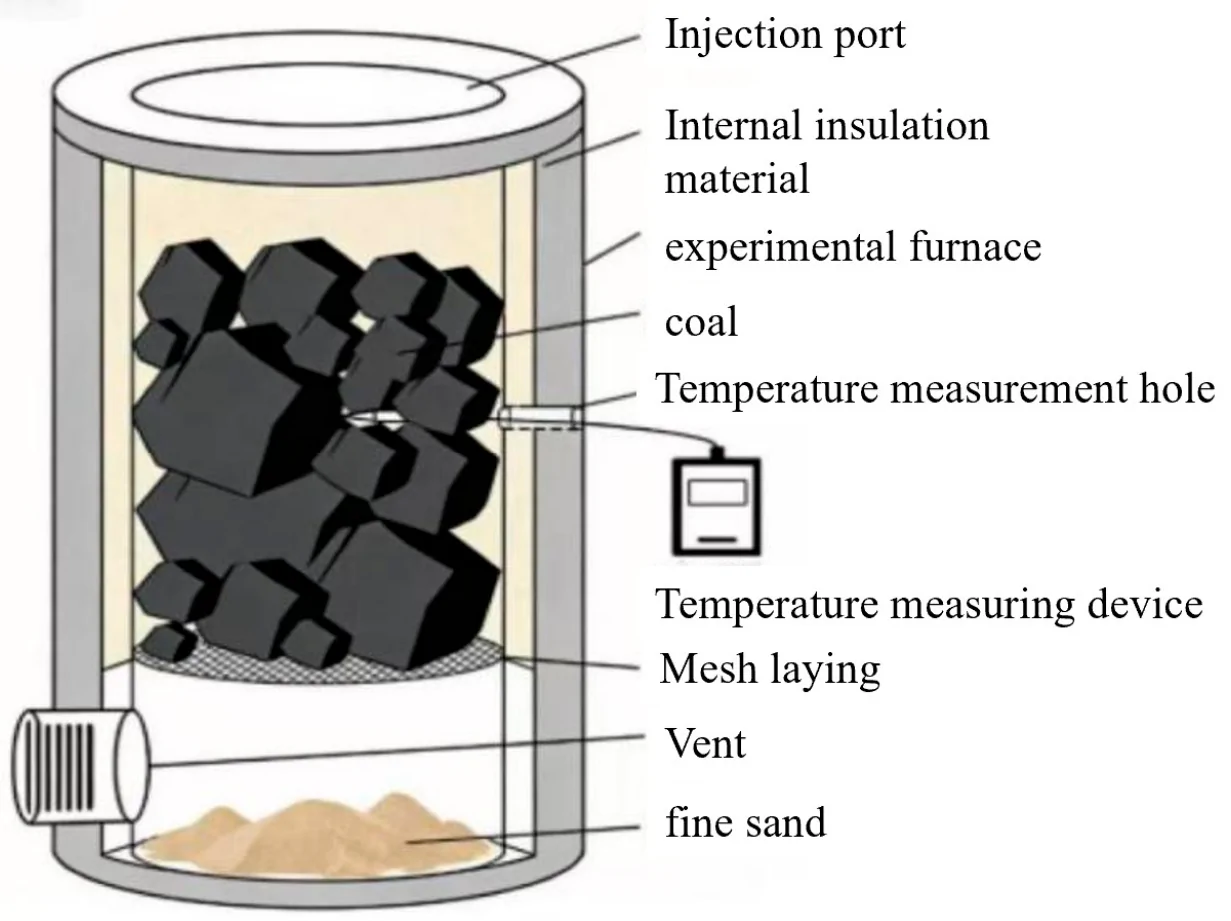
Schematic diagram of the fire-extinguishing test apparatus.

**Figure 14:**
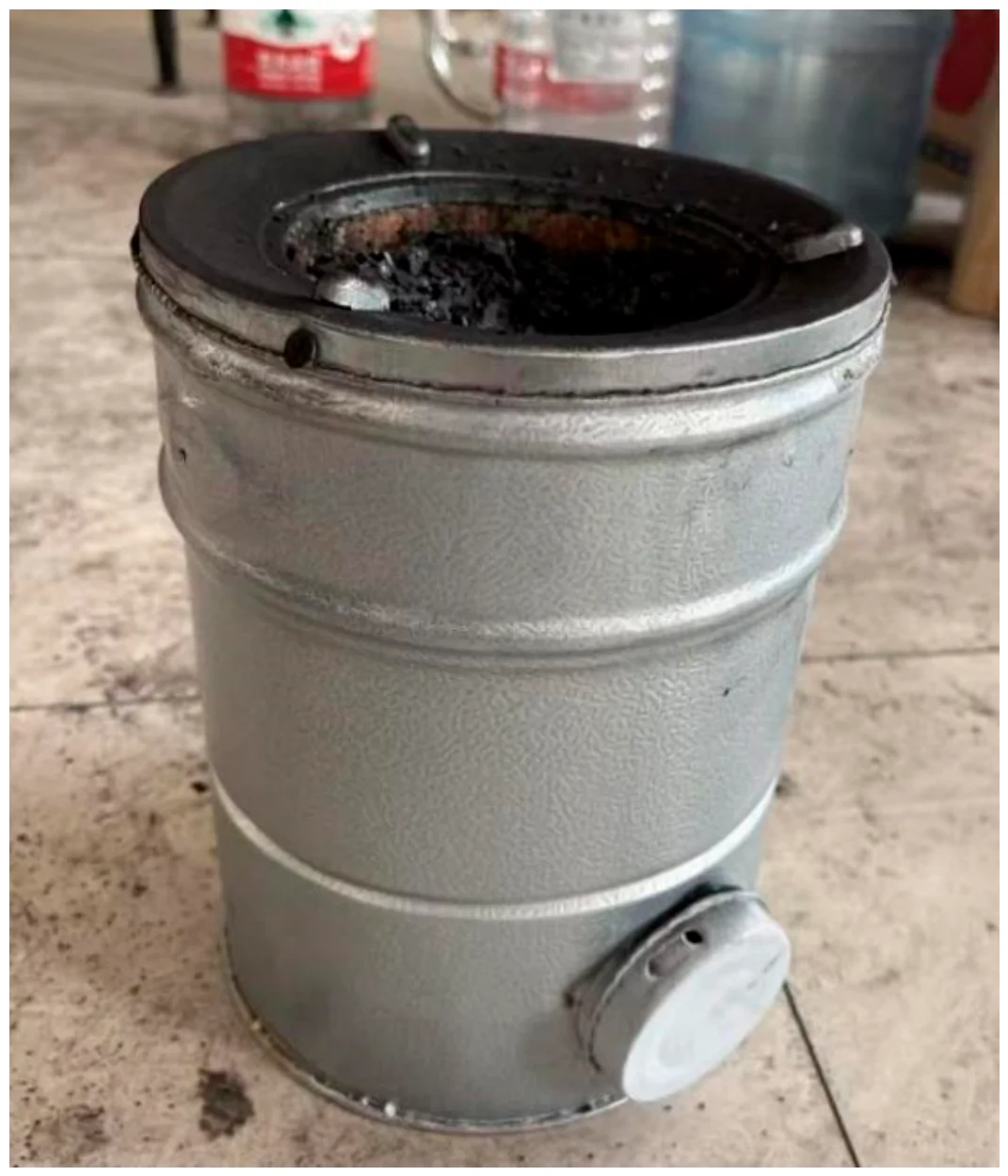
Actual photograph of the fire-extinguishing test apparatus.

A self-designed fire-extinguishing experimental device was used to test the performance of the flame-retardant dust-suppressant materials, which can effectively verify its application efficiency and adaptability in actual fire-extinguishing scenarios [31,32]. The device has an outer diameter of 20 cm, a height of 25 cm and an inner diameter of 15 cm. The cavity is filled with expanded perlite insulation material. A through-hole with a diameter of 0.5 cm is reserved in the middle of the cavity for the installation of temperature-sensing probes. A vent with a diameter of 5 cm is set at the bottom, and a fine sand layer with a thickness of 1 cm is laid. The experimental device can simulate the real coal spontaneous combustion condition, and comprehensively evaluate the fire-extinguishing rate and fire-extinguishing thoroughness by testing the coverage performance, permeability, cooling effect and oxygen insulation ability of the flame-retardant material. The schematic diagram of the fire-extinguishing experimental device and the physical diagram after the completion of the construction are shown in Figure 13 and Figure 14.

(1) Coal sample preparation

The coal samples used in the test were taken from the original coal seam of Shenmu Chuangwei Coal Mine.

In the sample preparation stage, the original coal sample was initially crushed by a Hubei crusher, and then the crushing product was screened to obtain five different particle sizes of 0~0.9 mm, 0.9~3 mm, 3~5 mm, 5~7 mm and 7~10 mm, a total of 2000 g of mixed particle size.

(2) Test material preparation

Test group 1: Prepare 5% (100 g) of clean water for standby;

Test group 2: Preparation of flame-retardant dust suppressant accounting for 5% (100 g) of coal sample mass, standby;

Test group 3: Preparation of flame-retardant dust suppressant accounting for 10% (200 g) of coal sample quality, standby.

(3) Experimental test

In the experiment, solid alcohol is used to ignite the coal body. At the same time, the blower is used to supply air to the interior of the experimental device through the vent to increase the temperature of the coal body. When the coal temperature rises to 800 °C, the air supply is stopped and the test material is added. The experimental temperature is read by a TM-902C digital thermometer. The experimental data are recorded every 10 s, and the test time is 0–400 s.

## Figures and Tables

**Table 1:** Orthogonal experiment results.

Experiment Number	CMC Content (%)	APP Content (%)	ZB Content (%)	PCDI Content (%)	Penetration Depth (cm)
1	1	8	2	0.5	35.2
2	1	12	3.5	1.5	27.5
3	1	16	5	2.5	20.6
4	1.5	8	5	1.5	22.5
5	1.5	12	2	2.5	15.8
6	1.5	16	3.5	0.5	32.6
7	2	8	3.5	2.5	9.4
8	2	12	5	0.5	23.3
9	2	16	2	1.5	17.2

**Table 2:** Industrial analysis results of test coal samples.

Coal	Industrial Analysis				
Bituminous coal	M_ad_ (%)	A_d_ (%)	V_daf_ (%)	S_t,d_ (%)	V_d_cm^3^/g
	3.21	3.32	33.12	0.33	0.82

**Table 3:** Performance comparison between CMC-based composite gels and typical traditional inhibitors.

Performance Metric	Water	Single CMC	Single APP/ZB	Physical Blend (APP + ZB)	CMC-PCDI-APP-ZB Gel (This Work)
Wetting (Contact Angle)	Slow (>5 s)	Moderate (~1 s)	Poor (insoluble)	Moderate (~0.8 s)	72.8° → 17.2° in 0.3 s
Wind Erosion Loss (30 min)	High (~15%)	Moderate (~10%)	High	Moderate (~8%)	4.16%
Ignition Temp. Increase	Limited (~5 °C)	Limited (~3 °C)	Moderate (+8/+5 °C)	Moderate (+10 °C)	+16.8 °C
CO Reduction at 170 °C	Low (~10%)	Low (~5%)	Moderate (+15%/+10%)	Moderate (+20%)	40%
Anti-Reignition (800 → 100 °C)	Severe (>600 °C)	Weak	Weak	Partial	None
Mechanism	Physical cooling	Physical wrap	Chemical inhibition	Unclear synergy	Network confinement + P-B synergy

## Data Availability

Date are contained within the article.
